# Identification of New Provisional Simian Adenovirus Species from Captive Monkeys, China

**DOI:** 10.3201/eid2010.131255

**Published:** 2014-10

**Authors:** Kimberly R. Foytich, Garland Deshazer, Mathew D. Esona, Angela Liu, Yuhuan Wang, Xinming Tu, Baoming Jiang

**Affiliations:** Centers for Disease Control and Prevention, Atlanta, Georgia, USA (K.R. Foytich, G. Deshazer, M.D. Esona, A. Liu, Y. Wang, B. Jiang);; Chinese Academy of Medical Sciences, Beijing, China (X. Tu)

**Keywords:** adenovirus, simian adenovirus, viruses, provisional species, China, monkeys

**To the Editor:** Adenoviruses commonly infect vertebrates, including humans and nonhuman primates (*1*). Adenoviruses from humans and animals were initially classified by differences in their agglutinating erythrocytes; now, however, they are often classified by sequence and phylogenetic analyses of their respective genes (*2*). One simian adenovirus species, SAdV-A, has been recognized by the International Committee on Taxonomy of Viruses (*2*). In addition, at least 25 simian adenovirus types have been identified (*3*). Since 2011, several novel adenoviruses have been isolated from nonhuman primates, including a group of new strains, designated SAdV-B, from asymptomatic rhesus macaques housed at 5 different primate facilities located across the United States (*2*,*4*).

Hexon is the most abundant protein in the icosahedral capsid, and the hexon gene is commonly analyzed to characterize and determine adenovirus types (*5*). We previously reported the detection of several new simian adenovirus strains in fecal specimens from captive monkeys in China (*3*). These novel strains were detected by using PCR and hexon gene–specific primers, but the product length (≈255 bp) may be insufficient to completely and accurately characterize these strains. Consequently, we further characterized 10 of these novel simian adenovirus strains by sequencing the entire open-reading frames of the hexon genes. Of the 10 strains, 9 were from rhesus macaque (CHN-8, CHN-14, CHN-23, CHN-30, CHN-36, CHN- 39, CHN-43, CHN-48, and CHN-51), and 1 was from a pigtail macaque (CHN-24).

We performed alignment analysis of the complete nucleotide hexon sequences of the 10 novel strains and of reference human and simian adenoviruses by using the MUSCLE program within the MEGA5 software package (*6*). To best fit the sequence data for the hexon gene, we used the DNA/Protein model test to identify the optimal evolutionary model, GTR+Γ+I (general time reversible + gamma + invariable). To construct the maximum-likelihood tree, we used the corrected Akaike Information Criterion and the MUSCLE program within MEGA5. Nucleotide and amino acid distance matrixes were prepared by using the *p*-distance algorithm of MEGA5.

Hexon genes of the 10 simian adenovirus strains ranged in length from 2,739 to 2,820 nt and from 913 to 940 aa. Four strains (CHN-8, CHN-39, CHN-43, and CHN-48) clustered with SAdV-A species, showing 81%–86% nt and 90%–93% aa identity (Figure). One strain (CHN-14) clustered with the newly identified SAdV-B species, showing 89% nt and 97% aa identity to strain SAdV-50. Two strains (CHN-30 and CHN-51) clustered with HAdV-G species, showing 87% nt and 93% aa and 85% nt and 94% aa identities, respectively, to strain HAdV-52. Three strains (CHN-23, CHN-24, and CHN-36) formed a new cluster that was not closely related to any of the previously reported strains. Comparison of the hexon gene sequences of simian adenovirus types identified in this study with human and simian adenovirus sequences from GenBank revealed 9 previously reported hypervariable regions (HVRs): HVR1–HVR9 (*5*,*7*). The simian adenovirus types within the same cluster were closely related in all 9 HVRs (data not shown).

**Figure:**
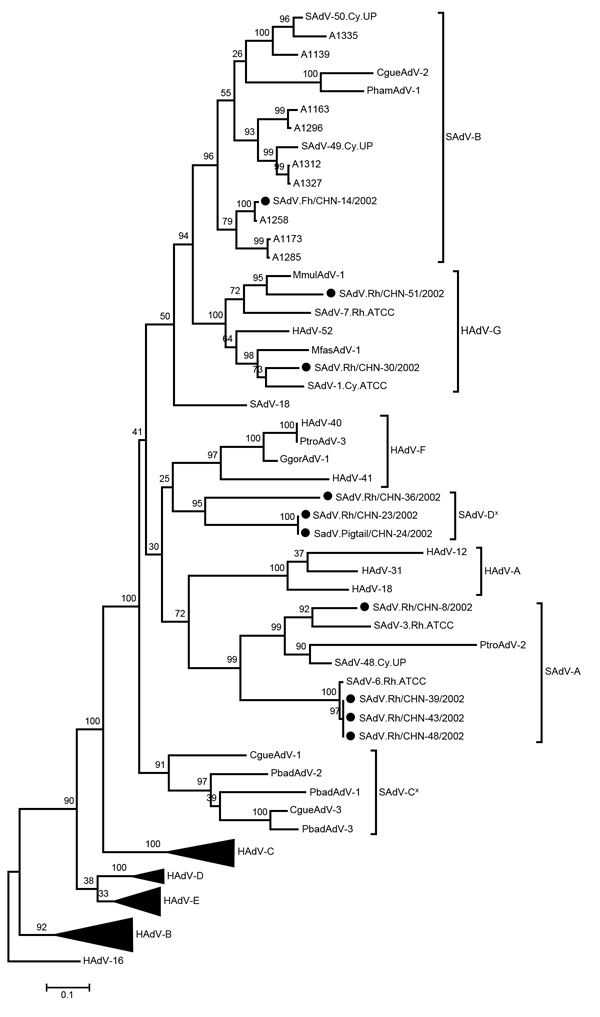
Phylogenetic analysis of complete hexon gene nucleotide sequences from human and simian adenoviruses. The maximum-likelihood tree was constructed as described in the text. Black dots indicate strains sequenced in this study; the sequences were deposited in GenBank (accession nos. KF053121–KF053130). Sequences for all reference strains used in the phylogenetic analysis were obtained from GenBank. Asterisks (*) indicate the proposed new candidate simian species. Numbers along branches and at nodes indicate bootstrap values. Scale bar indicates the branch lengths measured in the number of nucleotide and amino acid substitutions per site. AdV, adenovirus; ATCC, American Type Culture Collection; CHN, China; CgueAdV, *Colobus guereza* adenovirus; GgorAdV, *Gorilla gorilla gorilla* adenovirus; HAdV, human adenovirus; MfasAdV, *Macaca fascicularis* adenovirus; MmulAdV, *Macaca mulatta* adenovirus; PbadAdV, *Piliocolobus badius* adenovirus; Pham, *Papio hamadryas* adenovirus; PtroAdV, *Pan troglodytes schweinfurthii* adenovirus; and SAdV, simian adenovirus.

For more accurate characterization and for possible species determination of adenovirus strains, it is beneficial to sequence the entire hexon gene. In our previous study (*3*), partial hexon sequence analyses showed that strains SAdV.Rh/CHN-23/2002 and SAdV.Rh/CHN-36/2002 (formerly named 23 M.m. and 36 M.m., respectively) clustered with HAdV-G species, and strain SAdV.pigtail/CHN-24/2002 (previously named 24 M.n.) clustered with SAdV-A species. However, sequencing of the complete hexon gene showed that the 3 strains appeared to be distinct from other strains and formed a potentially new species cluster. Thus, we propose a new candidate simian species, named SAdV-D, for this new cluster of macaque adenovirus strains.

Our phylogenetic analysis also identified another possible new simian species: 5 strains, which were previously reported as “unassigned adenoviruses,” were isolated from Old World monkeys living in captivity and in the wild (*8*). We propose to designate this unassigned adenovirus cluster as candidate species SAdV-C (Figure). To definitively classify these species, further analyses of their entire genome sequences and organizations are needed. We also propose new strain designation nomenclature as follows: virus origin/isolation location plus strain identifier/isolation year. We believe that standard nomenclature and criteria are essential for identifying and classifying the increasing number of novel human and animal adenovirus strains that have been described on the basis of partial sequence data.

Our results demonstrated sequence conservation in the hexon genes of some adenovirus strains but also high diversity among other strains in a colony of captive monkeys. Our findings of close sequence relatedness between simian and human adenovirus strains suggest the interspecies transmission of these viruses and highlight the continuing risk for new and emerging infections in humans.
